# The accuracy of portion size estimation using food images and textual descriptions of portion sizes: an evaluation study

**DOI:** 10.1111/jhn.12878

**Published:** 2021-03-24

**Authors:** Desiree A. Lucassen, Romy F. Willemsen, Anouk Geelen, Elske M. Brouwer‐Brolsma, Edith J.M. Feskens

**Affiliations:** ^1^ Division of Human Nutrition and Health Wageningen University The Netherlands

**Keywords:** Dietary assessment, Food images, Household measures, Portion size estimation, PSEA, Standard portion sizes

## Abstract

**Background:**

Inaccurate self‐report of portion sizes is a major cause of measurement error in dietary assessment. To reduce this error, different portion size estimation aids (PSEAs) have been developed, including food images (image based, IB‐PSE) and textual descriptions of portion sizes (text‐based, TB‐PSE). We assessed the accuracy of portion size estimation by IB‐PSE and TB‐PSE.

**Methods:**

True intake of one lunch was ascertained in forty participants. Self‐reported portion sizes were assessed after 2 and 24 hours by means of TB‐PSE and IB‐PSE, in random order. Wilcoxon's tests were used to compare mean true intakes to reported intakes. Moreover, proportions of reported portion sizes within 10% and 25% of true intake were assessed. An adapted Bland‐Altman approach was used to assess agreement between true and reported portion sizes. Analyses were conducted for all foods and drinks combined and for predetermined food types.

**Results:**

No significant differences were observed between reported portion sizes at 2 and 24 hours after lunch. Combining median relative errors of all foods items resulted in an overall 0% error rate for TB‐PSE and 6% error rate for IB‐PSE. Comparing reported portion sizes within 10% (31% vs. 13%) and 25% (50% vs. 35%) of the true intake showed a better performance for TB‐PSE compared to IP‐PSE, respectively. Bland‐Altman plots indicated a higher agreement between reported and true intake for TB‐PSE compared to IB‐PSE.

**Conclusions:**

Although the use of TB‐PSE still results in measurement error, our results suggest a more accurate dietary intake assessment with TB‐PSE than IB‐PSE.

## Graphical abstract

Inaccurate self‐report of portion sizes is a major cause of measurement error in dietary assessment. To reduce this error, different portion size estimation aids have been developed. We assessed the accuracy of portion size estimation by using food images and textual descriptions of portion sizes.

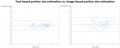

## INTRODUCTION

Accurate dietary assessment is essential in nutrition research. Although dietary intake is still often assessed using paper‐pencil tools, i.e. food frequency questionnaires (FFQs), food records (FRs) and 24‐hour recalls (24hRs), dietary assessment techniques have advanced rapidly in recent years. The last decade numerous valuable computer‐based and web‐based tools, mostly based on 24hRs and FFQs, have been developed.^1^, ^2^, ^3^ More recently also different smartphone applications (i.e. apps), mostly based on FRs, have been developed to collect real‐time dietary intake data.^3^, ^4^ Important benefits of these new tools include that they are assumed to lower burden on both participant and researcher compared to traditional techniques.^3^, ^4^, ^5^, ^6^

A fundamental aspect of accurate dietary assessment is portion size estimation.^6^, ^7^, ^8^ However, assessment of portion sizes is challenging and a major cause of error in dietary assessment.^6^, ^9^, ^10^, ^11^ Difficulties occur while reporting previously consumed foods as well as when judging displayed foods.^4^, ^11^, ^12^ The accuracy of portion size estimation is affected by various factors, including type of food and serving size.^6^, ^10^, ^13^ Generally, single‐unit foods (e.g. sliced bread, fruits) are more likely to be reported correctly compared to liquids or amorphous foods (e.g. pasta, lettuce).^4^, ^12^, ^14^ Another issue in portion size estimation is that large portions tend to be underestimated and small portions tend to be overestimated, which is also known as the ‘flat‐slope phenomenon’.^11^ In addition, foods consumed in small portions (e.g. spreads) are likely to be estimated more accurately than large portions of foods.^13^

Portion size estimation aids (PSEAs) (e.g. images, referent objects, portion size suggestions) have been suggested to result in more accurate portion sizes estimates.^15^, ^16^, ^17^ However, research indicates that these PSEAs still result in measurement error and that further optimization of PSEAs is needed ^17^, especially with respect to PSEAs that may be implemented in web‐based and smartphone‐based dietary assessment tools. The most commonly used PSEAs in web‐based and smartphone‐based tools are portion size suggestions (i.e. standard portion sizes and household measures), food images, and free entry of weight in grams.^1^ As individuals fail to recognize the metric quantities of portion sizes, estimations in grams are usually inaccurate.^18^ For this reason, participants tend to prefer the use of household measures rather than estimation in grams.^17^, ^18^ Yet, inconsistent or vague descriptions of household measures may still result in measurement error, especially among individuals that are not frequently involved in meal preparation.^18^, ^19^ Therefore, clear descriptions of the portion sizes are crucial.^20^

To facilitate the estimation of portion sizes, several dietary assessment tools have included food images as visual aids, where individuals are requested to select the most comparable image with respect to the portion size consumed or displayed (i.e. image‐based portion size assessment or IB‐PSE). Previous research indicates that IB‐PSE is particularly influenced by three main elements, namely perception, conceptualization and memory.^13^ Despite these elements of potential error, IB‐PSE is suggested to be a useful aid to estimate portion sizes.^14^, ^21^, ^22^, ^23^, ^24^ However, there is only limited evidence on the reliability of IB‐PSE in real‐life situations.^14^, ^19^ Up to now, the reliability of IB‐PSE has mainly been examined by exposing participants to foods and food images simultaneously while focussing on perception and not conceptualization and memory.^22^, ^23^, ^24^ More specifically, the majority of previous research only compared PSEAs to weighed portion sizes as a reference technique.^12^, ^19^, ^21^, ^22^, ^23^, ^24^ To the best of our knowledge, none of the previous studies examined the accuracy of portion size estimation using a combination of textual descriptions of household measures (e.g. spoons, cups, glasses), standard portion sizes (e.g. small, medium, large) and estimation in grams (i.e. for the purpose of this study referred to as text‐based portion size estimation or TB‐PSE) and IB‐PSE.

Therefore, the current study aimed to compare the accuracy of TB‐PSE and IB‐PSE. As we hypothesize that accuracy varies over different food types, accuracy of both PSEAs was examined for all foods and drinks combined and for specific food types. In addition, to gain a first insight in the effect of memory on the accuracy of the PSEAs, the portion sizes were reported after either 2 hours or 24 hours.

## MATERIALS AND METHODS

### Participants

Participants were recruited through a convenience sampling method using a database of research volunteers of the division of Human Nutrition and Health of Wageningen University and Research (WUR), social media accounts of the division (i.e. Facebook and Twitter), and through posters. Eligible participants were Dutch speaking, not visually impaired, not participating in another dietary intervention study, not an employee of the division, and not having any formal training in the field of nutrition. In total, 40 participants aged 20‐70 years old were included in this study that was conducted during a 2‐week period in February 2018. Participants were stratified by sex and age to ensure equal distribution of these characteristics and randomly assigned to two groups. Participants were informed that the study focused on different digital methods to assess food intake. The true study purpose was not disclosed until the end of the study. Written informed consent was obtained from all participants.

### Overall study design

Participants were invited for one lunch at the study centre as part of the cross‐over study and asked to complete two dietary questionnaires on a tablet or computer; 2 and 24 hours after lunch. The first group reported their food intake 2 hours after lunch by means of TB‐PSE and 24 hours after lunch by means of IB‐PSE. The second group reported their intake with the two PSEAs in the opposite order. As previous studies suggest that the potential difficulty to accurately estimate portion size depends on the type of food, we offered a variety of commonly consumed food types in the Netherlands ^7^, ^12^, ^13^, ^14^ (Table 1). Each participant was provided with pre‐weighed, ad libitum amounts of the food items. Each item was offered in a container without indication of the content. To minimize the effect of tableware on portion size estimation ^25^, the participants received a variety of tableware. After lunch, plate waste was weighed to assess true intake of each food item. Weights were taken with ‘Sartorius Signum 1’ calibrated weighing scales. True intake was calculated by the following formula:

$$\text{True}\;\text{intake}\;\left(g\right)=\text{Pre ‐ weighed}\;\text{food}\;\text{item}\;\left(g\right)‐\text{Plate}\;\text{waste}\;\text{food}\;\text{item}\;\left(g\right)$$

**Table 1:** Food items offered, by food type.

Offered food items
Amorphous ‐ Cheese ‐ Crunchy muesli ‐ Fruit salad ‐ Scrambled eggs ‐ Yogurt
Liquids ‐ Milk ‐ Orange juice ‐ Water
Single‐units ‐ Bread slices ‐ Bread rolls
Spreads ‐ Jam ‐ Margarine

### Portion size assessment

For the purpose of this study, a TB‐PSE and IB‐PSE questionnaire was developed in Qualtrics (Qualtrics, Provo, UT, USA). The question formulation and portion size estimation within the TB‐PSE questionnaire were based on Compl‐eat™; a self‐administered web‐based dietary 24hR‐tool developed by WUR ^20^. Portion sizes described in Compl‐eat™ are a combination of estimation in grams/millilitres, standard portion sizes and household measures, which are based on the ‘Food portion sizes and coding instructions’.^26^ The question formulation within the IB‐PSE questionnaire was also based on Compl‐eat™, thus ensuring that observed differences were solely due to the different PSEAs and not due to differences in question formulation. For the IB‐PSE questionnaire, the portion size images from the Automated Self‐Administered 24‐hour dietary recall (ASA24) picture book, developed by the National Cancer Institute, Bethesda, MD ^27^, were used. This picture book contains 3 to 8 portion size images per food item. To the best of our knowledge, this is the only freely available picture book portraying food images with known amounts (g) for research purposes.^28^ Questionnaires started with questions whether or not a type of food was consumed, which was followed by questions on the amount of food consumed by means of one of the PSEAs. An example question from each questionnaire can be found in Supplement S1.

### Additional measurements

On the study day, participants completed a short questionnaire about basic characteristics (i.e. age, sex, educational level). In addition, weight and height were measured to calculate participants’ BMI (kg/m^2^). Participants were characterized in three educational levels (low: primary or lower education, intermediate: secondary or higher vocational education, high: college or university) and four age groups (18‐28, 29‐45, 46‐55, 56‐70 years).

### Statistical analysis

Normally distributed data is displayed as means (M) and standard deviations (SDs) in case of continuous variables, or frequencies in case of categorical variables; non‐normally distributed data as medians and interquartile ranges (IQRs). Significant differences between true and reported intake, and between 2 and 24 hours, were assessed for each PSEA. To allow comparison between PSEAs across different food types, relative differences were calculated. As previous research indicated that accuracy of portion size estimation varies over food types, all analyses were conducted for all foods and drinks combined and for predetermined food types individually (i.e. “all foods excluding liquids”, “amorphous foods”, “liquids”, “single‐units”, “spreads”; Table 1). As there are no guidelines on the acceptable level of accuracy ^7^, ^14^, ^29^, the proportion of the reported intake that fell within 10% and 25% of true intake were assessed, which is in line with comparable studies in this research area.^14^ Proportions within 10% of true intake will be deemed acceptably accurate, whereas proportions within 25% of true intake will be used to get further insight in the levels of accuracy.^30^ To determine agreement between reported and true intake for both PSEAs, Bland‐Altman plots with 95% limits of agreement (LOA) were plotted. Usually the Bland‐Altman method is applied for assessing agreement between two imperfect measures. Since true intake was assessed an adapted Bland‐Altman method was used to plot the differences between reported and true intake against true intake.^14^, ^31^ However, when true intake increased, the absolute error increased. Therefore, we plotted the log‐transformed ratio of reported and true intake against log‐transformed true intake. Middle line indicates the mean and the upper and lower lines indicate borders based on mean ±1.96 SD. Since the variables were not normally distributed, Wilcoxon signed rank test was used to test within group and the Wilcoxon rank sum test was used to test for between group differences. All analyses were conducted with SAS software, version 9.4 (SAS Institute Inc., Cary, NC, USA). Statistical significance was set at *p* < 0.05.

## RESULTS

A total of 40 participants took part in this study. Participants had a mean ±SD age of 46.9 ± 19.2 years (range 20.7‐69.4 years), BMI 24.9 ± 3.8 kg/m^2^, 47.5% was men and the majority of the population was highly educated (62.5%). Participant characteristics did not significantly differ between group 1 (2hR: TB; 24hR: IB) and group 2 (2hR: IB; 24hR: TB) (Table 2). Furthermore, no significant differences were observed between reported at 2 and at 24 hours after lunch, for each PSEA. Therefore, the results are only shown per PSEA and are not subdivided per time point.

**Table 2:** Characteristics of the participants.

	Total (*n* = 40)			Group 1† (*n* = 20)			Group 2‡ (*n* = 20)		
	Mean	SD	%	Mean	SD	%	Mean	SD	%
Men			47.5			50.0			45.0
Age (years)	46.9	19.2		48.7	19.8		45.0	18.9	
BMI (kg/m^2^)	24.9	3.8		25.9	4.1		24.0	3.3	
Educational level									
Low			0.0			0.0			0.0
Intermediate			37.5			35.0			40.0
High			62.5			65.0			60.0

Note

No significant differences were found between groups.

† Group 1: 2hR = TB‐PSE; 24hR = IB‐PSE

‡ Group 2: 2hR = IB‐PSE; 24hR = TB‐PSE

Median true intake for “all foods and drinks combined” was 94 g (IQR: 128 g), while median reported intake was 75 g (IQR: 120 g) for TB‐PSE and 88 g (IQR: 164 g) for IB‐PSE. Comparing the true intake with the reported intake, as assessed with TB‐PSE, pointed towards significant differences for “all foods excluding liquids”, “amorphous foods”, “liquids” and “spreads” (Table 3). For IB‐PSE, significant differences with the true intake were observed for “all foods and drinks combined”, “liquids”, “single‐units” and “spreads”. For “all foods and drinks combined” the median relative difference was 0% (IQR: 44%) as assessed by TB‐PSE, and 6% (IQR: 115%) as assessed by IB‐PSE (Table 3).

**Table 3:** Median true intake, median reported intake for both PSEAs, and reported intakes within 10% and 25% of true intake for both PSEAs for all foods and per food type.

	Obs (n)	Median true intake (g, IQR)	TB‐PSE					IB‐PSE				
			Median rep. intake (g, IQR)	Median diff. (g, IQR)†	Median rel. diff. (%, IQR)‡	<10% of true (%)	<25% of true (%)	Median rep. intake (g, IQR)	Median diff. (g, IQR)†	Median rel. diff. (%, IQR)‡	<10% of true (%)	<25% of true (%)
All food items	326	94 (128)	75 (120)	0 (25)	0 (44)	31	50	88 (164)	3 (74)***	6 (115)***	13	35
All excl. liquids	263	66 (86)	50 (101)	0 (22)***	0 (44)	32	49	61 (95)	−1 (41)	−5 (74)**	15	41
Amorphous	150	84 (124)	66 (107)	−7 (45)**	−12 (56)	12	25	104 (119)	3 (70)	4 (109)***	13	31
Liquids	63	194 (125)	220 (150)	29 (80)***	15 (39)	30	56	355 (356)	211 (373)***	118 (122)***	5	11
Single‐units	59	100 (50)	100 (50)	0 (0)	0 (0)	95	95	72 (61)	−7 (25)*	−14 (18)	29	80
Spreads	54	17 (15)	15 (14)	−3 (14)*	−23 (63)	9	24	14 (12)	−3 (10)*	−22 (63)	9	30

Note

All food items., all foods and drinks combined; obs., observations; rep., reported; diff., difference; rel., relative; excl., excluding.

† Calculated as reported intake minus true intake. Thus, positive differences represent overestimations and negative differences represent underestimations. Significant differences between reported and true intake was assessed with a Wilcoxon signed‐rank test. Significant differences are indicated by * for *p* < 0.05, ** for *p* < 0.01, *** for *p* < 0.0001.

‡ Relative differences (%) = (reported intake (g) –true intake (g)) / true intake (g) * 100. Significant differences between intake reported with TB‐PSE and IB‐PSE were assessed with a Wilcoxon signed‐rank test. Significant differences are indicated by * for *p* < 0.05, ** for *p* < 0.01, *** for *p* < 0.0001 in the IB‐PSE “Mean rel. diff. column”.

Significantly higher relative errors were shown for IB‐PSE than for TB‐PSE for “all foods and drinks combined”, “all foods excluding liquids”, “amorphous foods” and “liquids”. For “all foods and drinks combined” the proportion of reported intakes within 10% of true intake was 31% for TB‐PSE and 13% for IB‐PSE, the proportion within 25% of true intake was 50% for TB‐PSE and 35% for IB‐PSE. For TB‐PSE, the lowest proportion within 10% and 25% of true intake was observed for “spreads”, whereas for IB‐PSE, the lowest proportion was observed for “liquids”. The highest proportion of reported intake that fell within 10% and 25% of true intake was, for both PSEAs, observed for the food type “single‐units” (Table 3).

The log‐transformed Bland‐Altman plot of “all foods and drinks combined” showed a higher level of agreement for TB‐PSE (M: 0.04; LOA: −1.11‐1.03) than for IB‐PSE, as shown by more widely scattered estimates and wider limits of agreement for IB‐PSE (Supplement S2). Excluding liquids did not substantially alter these findings; agreement for TB‐PSE (M: −0.10; LOA: −1.22‐1.00) remained higher compared to IB‐PSE (M:0.03; LOA: −1.37‐1.43). The same trend was observed for the other food types (Supplement S2). The highest level of agreement was observed for “single‐units” (TB‐PSE M: −0.02; LOA: −0.30‐0.25 vs. IB‐PSE M: −0.09; LOA: −0.84‐0.66), whereas the lowest level of agreement was observed for “amorphous foods” (TB‐PSE M: −0.13; LOA: −1.43‐1.15 vs. IB‐PSE M: 0.17; LOA: −1.38‐1.71).

## DISCUSSION

In this study, the reported intake and its estimation error for “all foods and drinks combined” using IB‐PSE significantly differed from true intake while no statistically significant difference was observed between the reported intake and its estimation error from true intake using TB‐PSE. However, as indicated by the proportion of reported intakes within 10% and 25% of true intake, being 31% and 50% using TB‐PSE compared to 13% and 35% using IB‐PSE, meaning that for both PSEA’s only the minority of estimations lies within the acceptable range, further improvements to increase the accuracy of portion size estimation are needed.

Before discussing our findings, the strengths and limitations of our study will be discussed. First, despite the fact that participants consumed their lunch in a controlled setting, we strived to mimic a real‐life situation. Specifically, in contrast to most other studies, participants could choose from a selection of food items and actually consumed the selected items.^19^, ^24^ Furthermore, participants had the opportunity to choose between different sizes of tableware ^25^ and had ad libitum access to the foods provided.^32^ Moreover, all products were served in bowls, jugs and plates without indication of content. Second, as the accuracy of two PSEAs was assessed separately, accuracy of both methods could be studied independently. Moreover, due to the study's cross‐over design the accuracy of both PSEAs was assessed in each participant. Third, to our knowledge, this is the first study comparing the two PSEAs, while keeping all other factors in the questionnaire identical. Finally, to avoid extra focus on portion sizes, participants were not informed on the goal of the study and did not see the weighing of the foods. A limitation of our study is that we used the ASA24 picture book in a Dutch population. The ASA24 is the only freely available photo database for research with known portion size weights. However, the ASA24 photographs are based on the 5^th^ and 95^th^ percentile of intake per product in the US and as such tailored for usage in the US.^14^, ^33^, ^34^ It is known that portion sizes in the US are larger than in the Netherlands.^35^, ^36^ To illustrate, the glasses in the study of Donders‐Engelen *et al*. ^26^ range between 100 g and 220 g whereas the glasses in ASA24 range between 177 g and 473 g. As ASA24 does not contain pictures of the smallest portion sizes consumed in the Netherlands, this may explain the overestimated intakes by IB‐PSE estimates in our study (e.g. 118% for “liquids”). However, we have to note that the portion size database that currently is being used in the Netherlands dates from 2003. It is known that plate sizes have increased in the past decades ^36^, which on its turn may have led to an underestimation of TB‐PSEs.

A more general limitation of the ASA24 food images is the usage of cutlery as reference, which is meant to help participants estimate the real‐life size of a portion. However, as cutlery can vary in size, it might not be the best reference and as such explain the more scattered points observed in the Bland‐Altman plot of IB‐PSE compared to TB‐PSE. Finally, in view of generalisability it needs to be mentioned that our participants were relatively old and highly educated. However, several previous studies concluded that age and education level did not affect the participants ability to estimate portion sizes.^19^, ^22^, ^23^, ^37^ In addition, we only tested a limited number of food items, and as such our findings are only applicable to these tested food items.

As hypothesized, the accuracy of reported intake with both PSEAs varied between the different food types. Both PSEAs overestimated the median reported intake of “liquids” whereas the intake of “all foods excluding liquids” and “spreads” were (slightly) underestimated. In addition, for TB‐PSE, the reported median intake of “amorphous foods” was underestimated, while for IB‐PSE the intake was overestimated. Previous research showed both under‐ and overestimations of portion size estimations.^7^, ^14^ Moreover, the accuracy of food intake estimates varied depending on the food types.^12^, ^13^, ^38^ Both PSEAs showed the highest estimation errors for “liquids, which is not in line with similar studies showing the highest estimation errors for “amorphous foods”.^12^, ^13^, ^14^, ^37^ In contrast to previous studies, which mostly provided liquids in containers that were identical to containers portrayed on the images, we aimed to resemble the real‐life situation and therefore studied commonly‐used PSEA descriptions and used glasses that did not necessarily match with the glasses on the images. As conceptualization plays a major role in the accurateness of portion size estimation^13^, it is easier to estimate portion sizes when the portion sizes are similar to the portions portrayed on the images^23^, ^39^ or the textual descriptions.^18^, ^20^ For instance, the description “lemonade glass” lacks detail and can easily result in misclassification. In agreement with our study, Hernandez *et al*. ^7^ also studied the intake of liquids in containers that were not identical to the containers on the images and also observed the highest estimation errors for liquids, which underlines the influence of conceptualization.

As illustrated by small errors for “single‐units” and “spreads” and larg(er) errors for “amorphous foods” and “liquids” for both PSEAs, our findings clearly indicate that foods consumed in small or defined units are more accurately estimated than foods consumed in larger amounts. These findings are in line with previous studies.^23^, ^37^, ^39^ Generally, the accuracy for the food types “amorphous foods”, “liquids” and “single‐units” was higher for TB‐PSE than for IB‐PSE estimates, except for “spreads” which were more accurately estimated with IB‐PSE. The latter may relate to the fact that textual description of the size of spoons and spread on bread is open to interpretation, whereas a picture may provide a better impression of the portion size estimate.^13^ Moreover, the fact that we used images of spoons, instead of images of spread on bread, to estimate the amount of “spreads” consumed, may have resulted in more accurate estimates for this food type.^12^ The size of the bread might influence the perception of the portion size and thereby lead to errors in estimations.^21^

We found no significant differences in accuracy between reporting after 2 hours and 24 hours for each of the PSEAs. Based on this, we concluded that memory did not influence the accuracy of portion size estimations within this timeframe. Therefore, only the combined results per PSEA were used for further analysis. However, after dividing the participants per PSEA over the two time points, the sample size per group was very small (i.e. ~20 participants) and therefore we had less power to detect significant differences. Previous research has shown that errors increase after 1‐2 hours, compared to immediate estimations.^24^ However, our first time point was after two hours and in line with our results, De Keyzer *et al*. ^21^ found no increase in estimation errors after 1‐2 days compared to after 4 days.^21^ To truly understand the effect of memory on accuracy of portion size estimation more research is needed with a larger sample size.

Due to lack of consensus on the minimal required level of accuracy for PSEAs no strong conclusion can be drawn on that matter. However, the accuracy of the reported intake by TB‐PSE was higher than by IB‐PSE for all food types except for “spreads”, which was higher with IB‐PSE. Overall, TB‐PSE provided more accurate portion size estimations than IB‐PSE. As discussed, these findings are different from previous studies.^14^, ^21^, ^22^, ^23^, ^24^ However, in contrast to these studies we incorporated all elements that influence IB‐PSE (i.e. perception, conceptualization, memory), instead of focusing on one or two of these elements ^22^, ^23^, ^24^, in an attempt to mimic a real‐life situation. Therefore, our findings in combination with previous studies may indicate that IB‐PSE is a useful PSEA, but only when judging displayed foods and not for retrospective portion size estimation.

TB‐PSE and IB‐PSE were selected due to their applicability for implementation in web‐based and smartphone‐base dietary assessment tools. However, there are other PSEAs which would be applicable for implementation in web‐based or smartphone‐based dietary assessment tools (e.g. remote food photography method, body‐worn monitors).^8^, ^40^ These innovative tools also have a range of drawbacks, for instance, it is known that they are unable to detect all aspects of the food consumed (e.g. no difference detected between spinach vs. spinach a la crème).^41^ Furthermore, individuals might feel uncomfortable wearing the device, especially long‐term, and it is difficult to guarantee the privacy of bystanders.^40^ Moreover, even though these devices have been proven to be up to 90% accurate^40^, such devices are expensive and therefore not suited for large‐scale studies. Selecting a PSE‐tool needs to be considered carefully while taking into account study design, methods and target group.^8^ Therefore, even though there are new, more innovative PSE‐tools being developed, it is still valuable to further improve both TB‐PSE and IB‐PSE. These PSEAs are easy to implement in web‐based and smartphone‐based tools, relatively inexpensive, well‐known and therefore easy to use with limited training.

To conclude, in our study TB‐PSE is shown to be more accurate than IB‐PSE. Country‐specific pictures with a clear reference are needed to improve the accuracy of IB‐PSE. Next to this, we can conclude that TB‐PSE seems to be an accurate PSEA for “single‐units”, as 95% of the reported intake fell within 10% of true intake. However, for the other food types, only 32% or less of the reported intakes fell within 10% of truth. Therefore, in line with Bucher *et al*. ^42^, we conclude that the accuracy of portion size estimations with TB‐PSE needs to be improved further and therefore standardized terminology is needed to avoid ambiguity with regard to textual descriptions of portion sizes. Finally, the use of a combination of PSEAs might be valuable to increase accuracy of portion size estimation.

## ETHICS STATEMENT

According to the Central Committee on Research involving Human Subjects (CCMO), this type of study did not require approval from an ethics committee in the Netherlands.

## CONFLICT OF INTEREST

The authors had no financial or personal conflicts of interest to declare.

## AUTHOR CONTRIBUTION

The authors contributions are as follows: D.A.L. contributed to the study design, data collection, interpretations of the findings, data analysis and drafted the manuscript; R.F.W. contributed to the study design, data collection, data analysis, interpretation of the findings and revised earlier versions of the manuscript; A.G. contributed to the study design, interpretations of the findings and revised earlier versions of the manuscript; E.B.B. and E.J.M.F. contributed to the interpretations of the findings and revised earlier versions of the manuscript. All authors read and approved the final version of the manuscript.

## TRANSPARENCY DECLARATION

The lead author affirms that this manuscript is an honest, accurate, and transparent account of the study being reported. The reporting of this work is compliant with STROBE guidelines. The lead author affirms that no important aspects of the study have been omitted and that any discrepancies from the study as planned have been explained. The study has not been registered in any trials registry.

### Peer Review

The peer review history for this article is available at https://publons.com/publon/10.1111/jhn.12878.

## Supporting information

Supplement S1

Supplement S2
