# Supplementary material for: The accuracy of portion size estimation using food images and textual descriptions of portion sizes: an evaluation study
Source: J Hum Nutr Diet. 2021 Mar 24;34(6):945–52. doi: 10.1111/jhn.12878 (PMC9291996; doi:10.1111/jhn.12878)
Supplement: Supplementary file 1 — Supplement S1 [file JHN-34-945-s001.docx]

# Supplement A

*Example question of TB-PSE questionnaire*

**Scrambled eggs:** How much did you eat (first select a serving unit and then insert the number of servings)?

| Grams (1 gram) |
| --- |
| 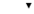Grams (1 gram) |
| Pieces (50 grams)  Tablespoon (15 grams) |

Insert the number of consumed servings for **scrambled eggs**:

|  |
| --- |

*Example question of IB-PSE questionnaire*

**Scrambled eggs:** How much did you eat (first select a serving unit and then insert the number of servings)? (4 images)


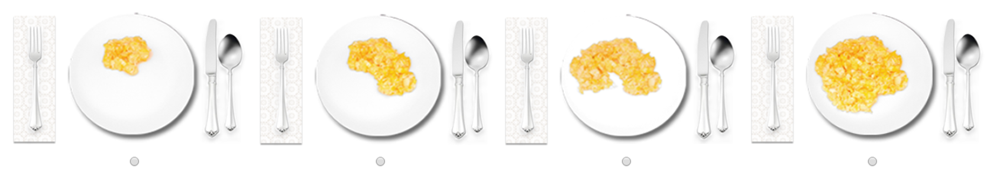
 Insert the number of consumed servings for **scrambled eggs**:

|  |
| --- |
