# Supplementary material for: The accuracy of portion size estimation using food images and textual descriptions of portion sizes: an evaluation study
Source: J Hum Nutr Diet. 2021 Mar 24;34(6):945–52. doi: 10.1111/jhn.12878 (PMC9291996; doi:10.1111/jhn.12878)
Supplement: Supplementary file 2 — Supplement S2 [file JHN-34-945-s002.docx]

# Supplement B

## All foods and drinks


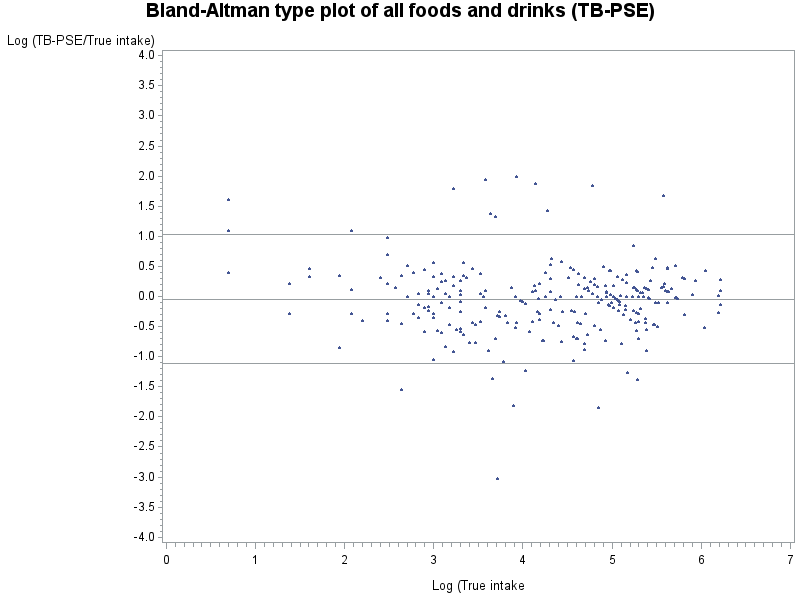


**Figure 1.** Bland-Altman plot of log transformed proportion of TB-PSE/true intake against log transformed true intake with mean proportion and 95% limits of agreement as reference lines for all foods and drinks.


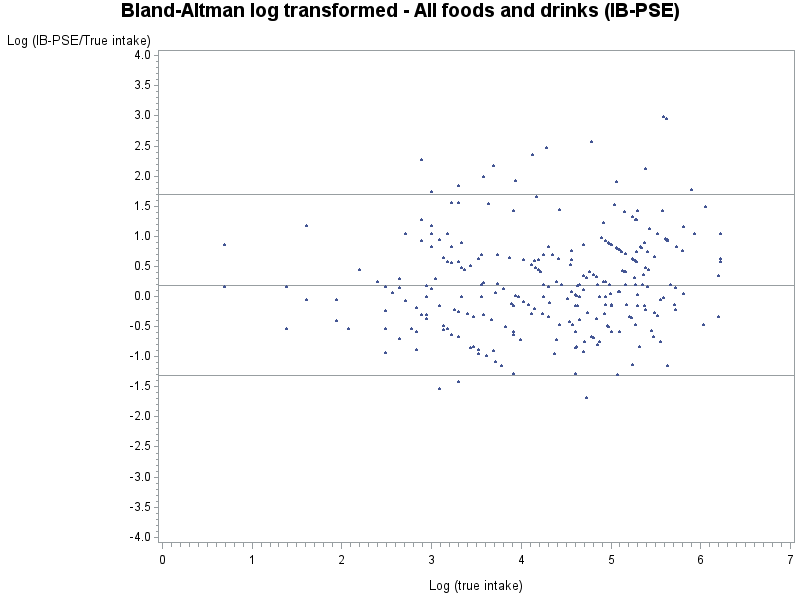


**Figure 2.** Bland-Altman plots of log transformed proportion of IB-PSE/true intake against log transformed true intake with mean proportion and 95% limits of agreement as reference lines for all foods and drinks.

## All foods excluding liquids


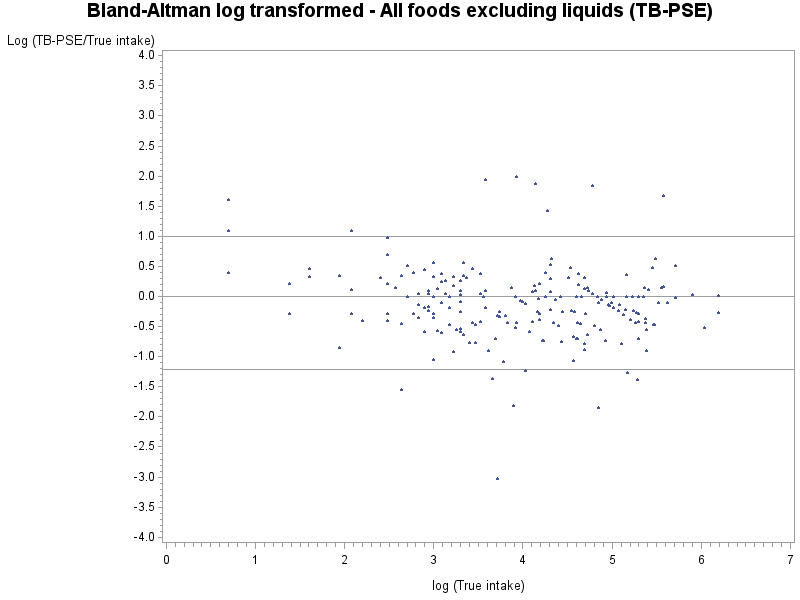


**Figure 3.** Bland-Altman plot of log transformed proportion of TB-PSE/true intake against log transformed true intake with mean proportion and 95% limits of agreement as reference lines for all foods excluding liquids.


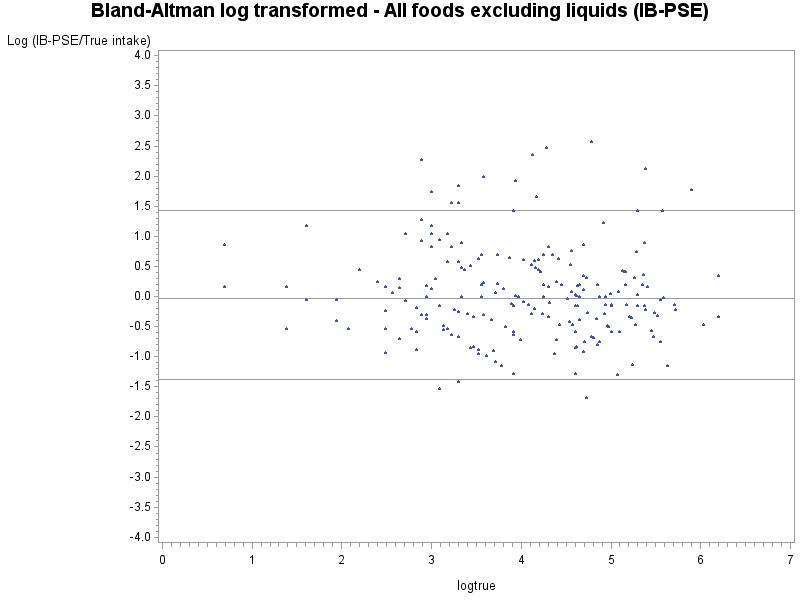


**Figure 4.** Bland-Altman plots of log transformed proportion of IB-PSE/true intake against log transformed true intake with mean proportion and 95% limits of agreement as reference lines for all foods excluding liquids.

## Amorphous foods


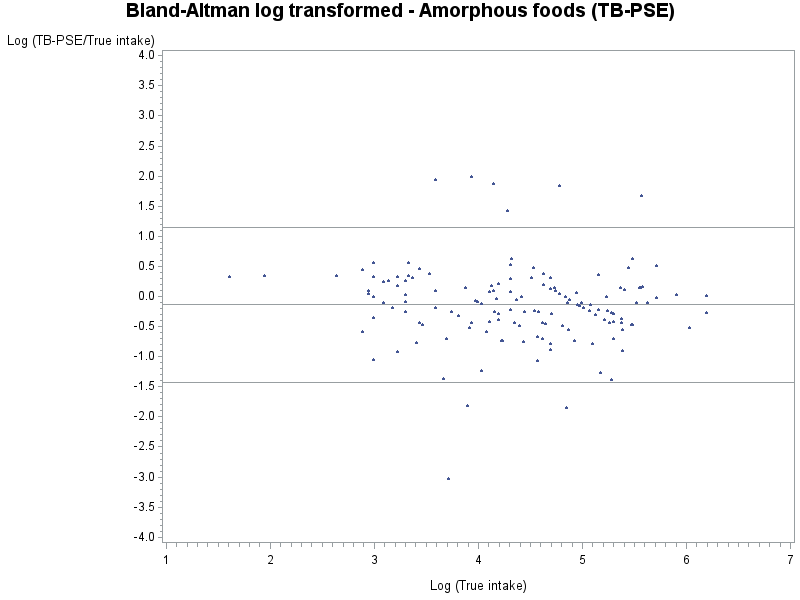


**Figure 5.** Bland-Altman plot of log transformed proportion of TB-PSE/true intake against log transformed true intake with mean proportion and 95% limits of agreement as reference lines for amorphous foods.


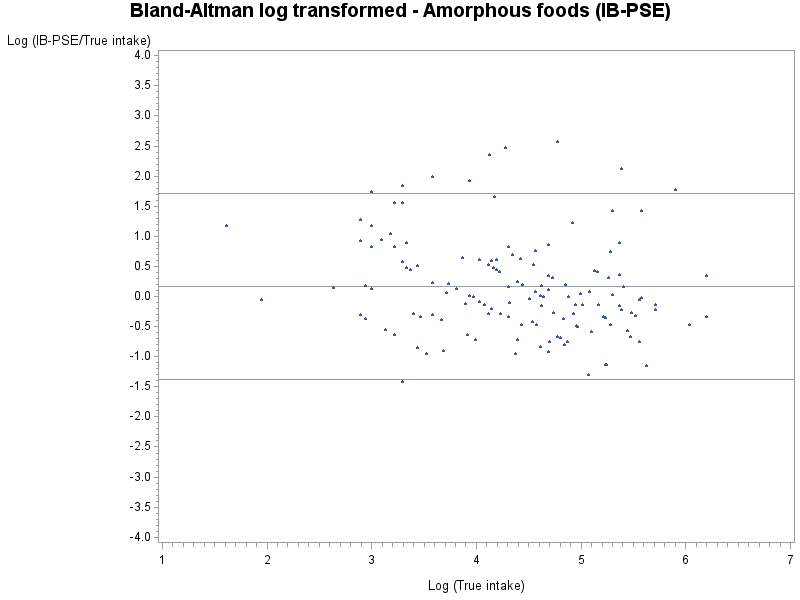


**Figure 6.** Bland-Altman plots of log transformed proportion of IB-PSE/true intake against log transformed true intake with mean proportion and 95% limits of agreement as reference lines for amorphous foods.

## Liquids


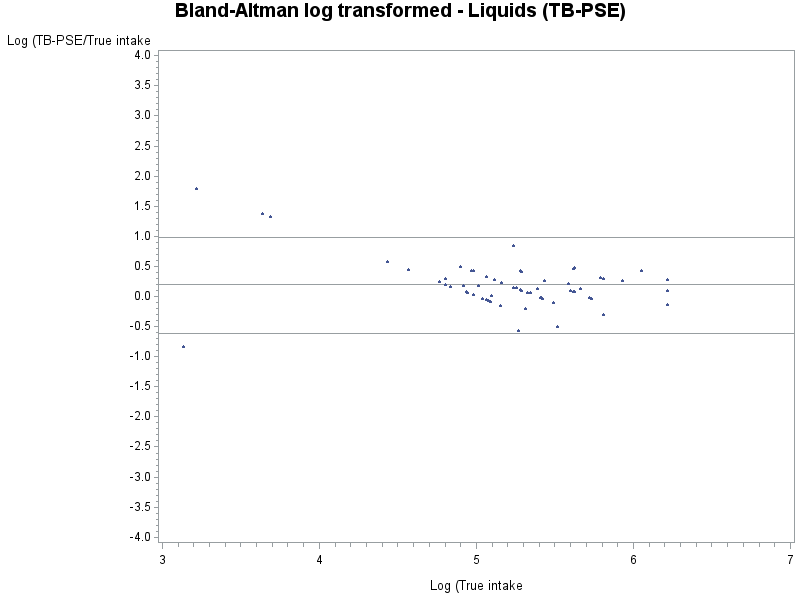


**Figure 7.** Bland-Altman plot of log transformed proportion of TB-PSE/true intake against log transformed true intake with mean proportion and 95% limits of agreement as reference lines for liquids.


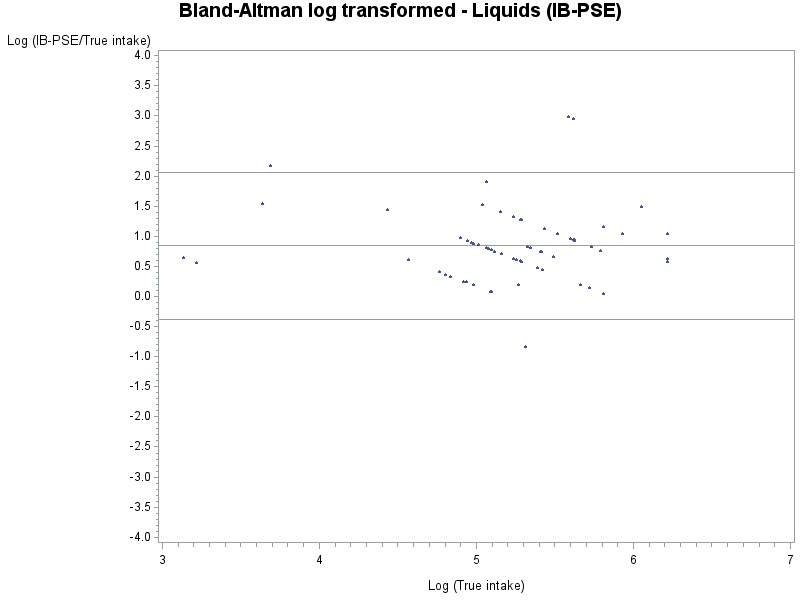


**Figure 8.** Bland-Altman plots of log transformed proportion of IB-PSE/true intake against log transformed true intake with mean proportion and 95% limits of agreement as reference lines for liquids.

## Single-unit foods


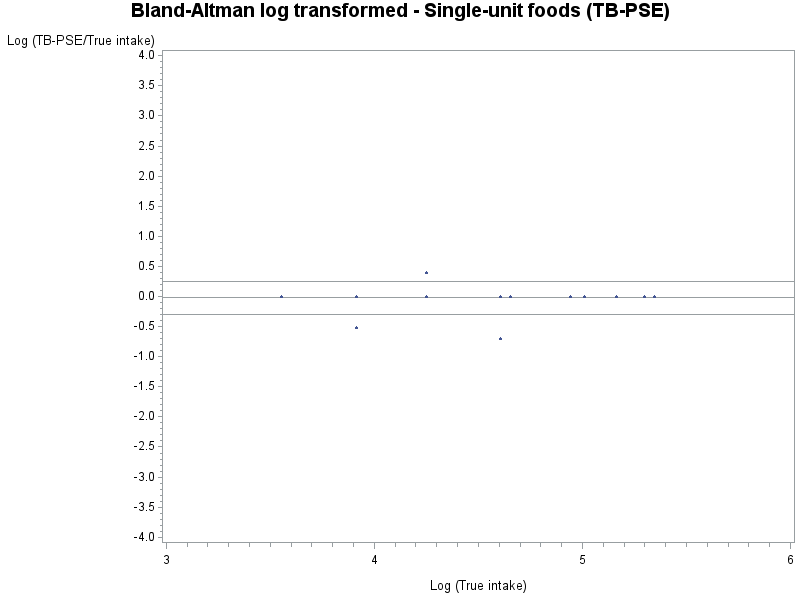


**Figure 9.** Bland-Altman plot of log transformed proportion of TB-PSE/true intake against log transformed true intake with mean proportion and 95% limits of agreement as reference lines for single-unit foods.


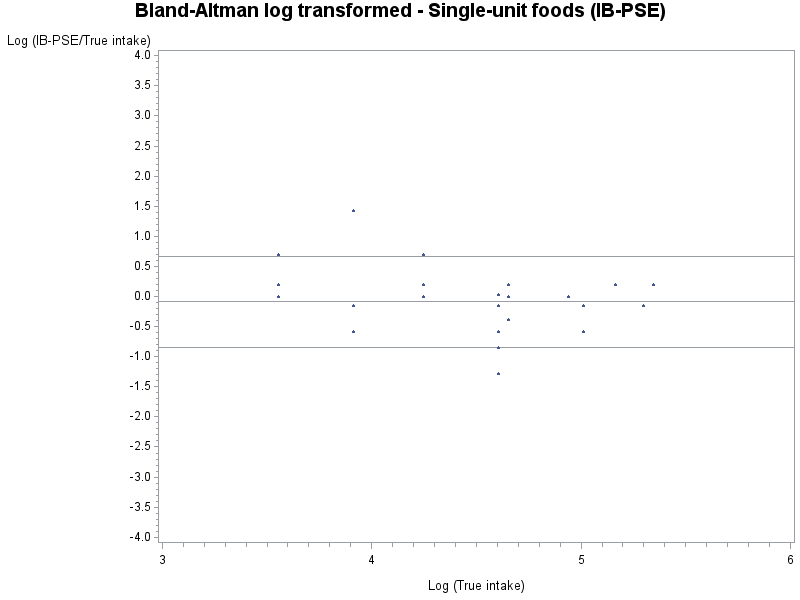


**Figure 10.** Bland-Altman plots of log transformed proportion of IB-PSE/true intake against log transformed true intake with mean proportion and 95% limits of agreement as reference lines for for single-unit foods.

## Spreads


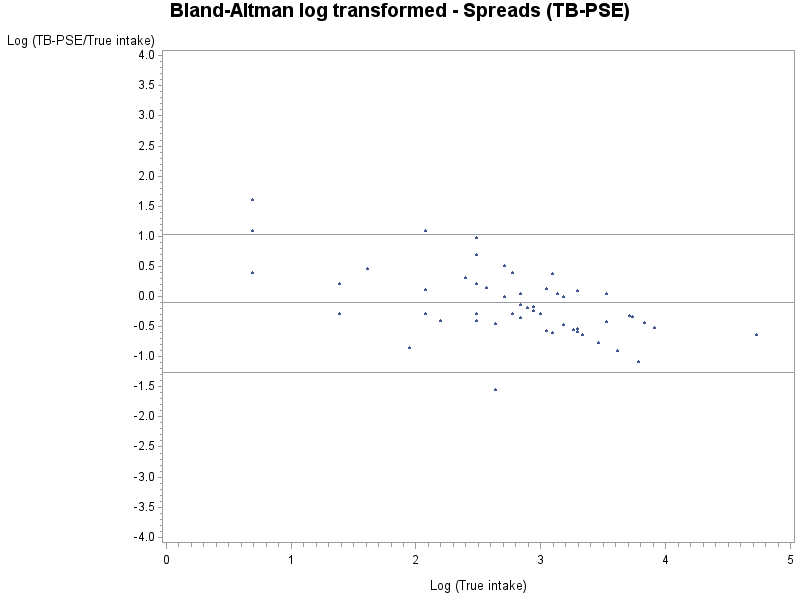


**Figure 11.** Bland-Altman plot of log transformed proportion of TB-PSE/true intake against log transformed true intake with mean proportion and 95% limits of agreement as reference lines for spreads.


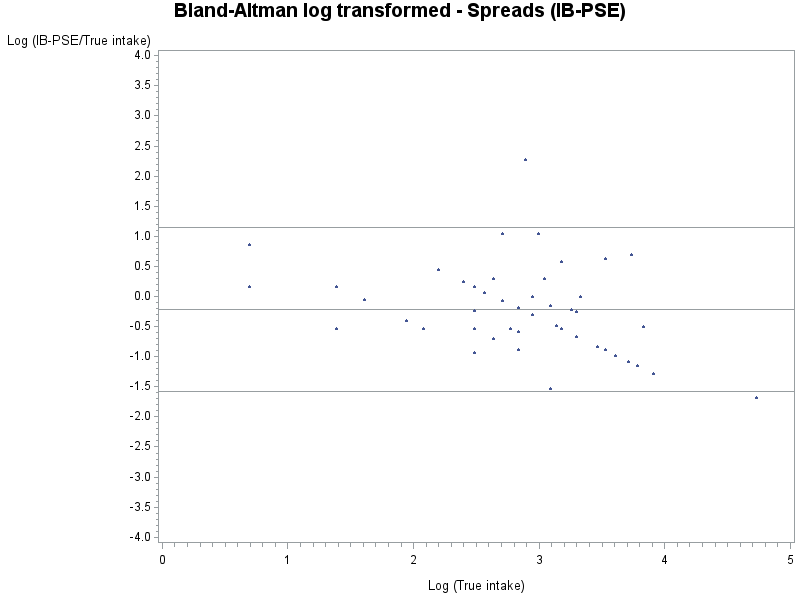


**Figure 12.** Bland-Altman plots of log transformed proportion of IB-PSE/true intake against log transformed true intake with mean proportion and 95% limits of agreement as reference lines for for spreads.
